# Packages of Care for Attention-Deficit Hyperactivity Disorder in Low- and Middle-Income Countries

**DOI:** 10.1371/journal.pmed.1000235

**Published:** 2010-02-23

**Authors:** Alan J. Flisher, Katherine Sorsdahl, Sean Hatherill, Sonia Chehil

**Affiliations:** 1 Division of Child and Adolescent Psychiatry, Adolescent Health Research Unit, University of Cape Town, Rondebosch, Republic of South Africa; 2 Department of Psychiatry and Mental Health, University of Cape Town, Republic of South Africa; 3 Division of Child and Adolescent Psychiatry, Dalhousie University, Canada

## Abstract

In the sixth in a series of six articles on packages of care for mental disorders in low- and middle-income countries, Alan Flisher and colleagues discuss the treatment of attention-deficit hyperactivity disorder.

*This is the last in a series of articles highlighting the delivery of “packages of care” for mental health disorders in low- and middle-income countries. Packages of care are combinations of treatments aimed at improving the recognition and management of conditions to achieve optimal outcomes.*

**Summary Points**

- Attention-deficit/hyperactivity disorder (AD/HD) is a multidimensional disorder that, although commonest in childhood and adolescence, can be diagnosed across the age span. Worldwide prevalence is about 5%.
- An appropriate package of treatment for AD/HD in low- and middle-income countries (LMICs) should include screening of high-risk groups, psychoeducational interventions with caregivers, methylphenidate, and behavioral interventions.
- Strategies to facilitate the delivery of effective interventions in LMICs should increase demand for services, access to AD/HD interventions, and the capacity of health care teams, as well as improve recognition of AD/HD, develop community-based and practice-based programs, and address the impact of AD/HD on other health and social outcomes.
- Interventions to address AD/HD should be part of a more comprehensive package of services for mental disorders.

## Introduction

Attention-deficit hyperactivity disorder (AD/HD) is a chronic, pervasive developmental disorder that, although usually diagnosed in childhood, spans the preschool to adult years. The most recent version of the Diagnostic and Statistical Manual of Mental Disorders (DSM-IV-TR) defines the disorder using the core features of age-inappropriate hyperactivity, impulsivity, and inattention (Box 1) [1]. The 10th edition of the International Classification of Diseases (ICD-10) provides operational criteria for the similar, but more severe and narrowly defined hyperkinetic disorder (HKD) [2]. We have used the term AD/HD throughout this paper because most of the published literature relates to the broader concept of AD/HD rather than to HKD.

**Box 1. DSM-IV-TR Criteria for Attention Deficit Hyperactivity Disorder and International Classification of Diseases 10 Criteria for Hyperkinetic Disorder**

According to the DSM-IV-TR, to satisfy the criteria for AD/HD, a patient must manifest six or more symptoms of inattention or hyperactivity-impulsivity that have persisted for at least six months “to a degree that is maladaptive and inconsistent with developmental level.” Examples of symptoms of inattention include often having difficulty in sustaining attention in tasks or play activities, being easily distracted by extraneous stimuli, and often being forgetful in daily activities. Examples of symptoms of hyperactivity–impulsivity include often fidgeting with hands or feet or squirming in seat, often talking excessively, and often having difficulty awaiting turn. In addition, some of the symptoms causing impairment must have been present before the age of seven years, some impairment from the symptoms must be present in two or more settings, there “must be clear evidence of clinically significant impairment in social, academic, or occupational functioning,” and the symptoms should “not occur exclusively in the course of a Pervasive Developmental Disorder, Schizophrenia, or other Psychotic Disorder and are not better accounted for by another mental disorder” [1].

According to the ICD-10, to satisfy the criteria for hyperkinetic disorder, a patient must demonstrate “abnormality of attention, activity and impulsivity at home, for the age and developmental level of the child,” as evidenced at least three of a set of attention problems, plus at least three of a set of activity problems, plus at least three of a set of impulsivity problems. In addition, the patient must demonstrate “abnormality of attention at school or nursery (if applicable), for the age and developmental level of the child,” as evidenced by at least two of a set of attention problems and at least three of a set of activity problems. Also, there must be “directly observed abnormality of attention or activity,” which is “excessive for the child's age and developmental level.” Finally, the child must not meet criteria for selected other conditions, onset must be before the age of seven years, the duration must be at least six months, and the IQ must be above 50 [2].

A rapidly expanding body of literature from low- and middle-income countries (LMICs) largely refutes the notion that AD/HD is a “Western” concept, although cultural factors clearly influence illness perceptions and help-seeking behavior [3]–[7]. A systematic review and meta-regression analysis of 102 studies from all continents concluded that the worldwide pooled prevalence of AD/HD is 5.3%, and that the geographic variability between AD/HD prevalence estimates is best explained by the methodological characteristics of the studies [8].

A strong body of evidence from high-income countries (HICs) suggests that AD/HD is a neurobiological syndrome with complex genetic factors primarily implicated in its etiology. Although individual risk alleles identified by molecular genetic studies increase the risk of AD/HD only slightly, the mean estimate of total heritability is just under 80% [9]. A wide range of social determinants significantly influence the symptomotology of AD/HD in HICs. These include low socioeconomic status, low parental education, family conflict, parental mental disorder, severe early deprivation, and institutional upbringing [9]–[11]. The involvement of these multiple determinants in the symptomatology of AD/HD is consistent with the hypothesis that AD/HD is an etiologically heterogeneous, final common pathway disorder that is influenced by genes, environment, and gene–environment interactions. Other nongenetic causes of AD/HD identified in HICs are factors that affect early brain development, such as perinatal stress, low birth weight, prenatal smoking and alcohol use, obstetric complications, head injury, epilepsy, and HIV/AIDS [9]–[11]. The mediating and moderating influence of these variables on the development of AD/HD amongst children in LMICs is less well researched and does not always match with that seen in HICs [11].

## The Evidence on the Treatment of AD/HD

### Detection

Accurate detection and diagnosis of AD/HD is crucial for the effective management of individuals with the disorder [12]–[14]. Two structured approaches are available to detect and diagnose AD/HD: clinical diagnostic interviews and rating scales (Table 1). The only clinical diagnostic interview that we are aware has been assessed in LMICs is the Diagnostic Interview Schedule for Children (DISC-IV) [15], which has been validated for use in China [16] and for Xhosa-speaking South Africans [17]. In China, the test-retest reliability of the AD/HD component was excellent for the version of the interview given to parents (*k* = 0.81) and poor for the version given to adolescents (*k* = 0.25). Similarly, for the South African Xhosa version of the DISC-IV the parent version resulted in substantial reliability (*k* = 0.56) whereas the youth version resulted in poor reliability (*k* = 0.23). Although systematic reviews have investigated the numerous rating scales developed to diagnose AD/HD in HICs [12]–[14], only a few scales have been developed for use in LMICs. In Brazil, the test-retest reliability of a scale for use by teachers based on DSM-III-R diagnostic criteria ranged from 0.56 to 0.70 [18], and the diagnostic performance of the Child Behavior Checklist Attention problem scale resulted in moderate areas under the curve (AUC = 0.78) [19].

**Table 1:** The evidence in support of AD/HD treatment.

Intervention	Evidence from HICs	Evidence from LMICs
Screening and diagnosis	• Test-retest reliability of the Diagnostic Interview Schedule for Children (DISC-IV) [15]• Systematic review of measures used to diagnose AD/HD in preschool children [14]• Summary of evidence-based assessment of AD/HD [13]• Ten-year review of rating scales for assessing AD/HD [12]	• Test-retest reliability of the Chinese [16] and Xhosa [17] version of the DISC-IV.• Test-retest reliability of a scale developed for use by teachers to assess AD/HD in Brazilian schools [18]• Assessment of the Child Behavior Checklist-Attention problem scale in Brazil [19]
Methylphenidate	• Systematic reviews [20]–[23]	—
Behavioral therapy	• Review of evidence-based psychosocial treatments for AD/HD [47]• Meta-analyses of the efficacy of behavioral therapy for AD/HD [48]	—
Cognitive behavioral therapy	• Review of cognitive training for AD/HD children [50]• Review of cognitive, cognitive behavioral, and neural-based Interventions [49]	—
Family therapy	• Meta-analysis of parent involvement in treating AD/HD [52]• Cochrane Review of family therapy for AD/HD [51]	• Evaluation of Parent Management Training in Iran [53]• Evaluation of effectiveness of providing parents with training about attention enhancing tasks and child behavior in India [54]

### Medication

The efficacy of pharmacological interventions for the treatment of AD/HD in HICs is well established (Table 1) [20]–[23]. Although there are few studies on medication for AD/HD from LMICs, efficacy data from studies conducted in HICs are likely to be applicable to these settings. Pharmacologic agents used in the treatment of AD/HD include the psychostimulants (methylphenidate or amphetamine), atomoxetine, bupropion, tricyclic antidepressants (TCAs), and alpha-agonists [24],[25]. The psychostimulants, which have been used clinically for more than 50 years, are supported by the most robust efficacy and safety data and are available as affordable generic immediate and sustained release formulations [25]. Methylphenidate has been established as an effective short-term treatment for school-age children and adults with AD/HD, and there are some data, albeit sparse, that support its efficacy in preschoolers [26],[27]. The short-term efficacy of atomoxetine, a newer medication, is also supported by numerous large-scale clinical trials, although direct comparison studies with the psychostimulants have demonstrated greater treatment effect sizes for methylphenidate [28],[29]. Also, the psychostimulants are less expensive than atomoxetine and more likely to be available in LMICs. Longer-term open label studies in HICs for methyphenidate and atomoxetine support the efficacy and safety of both these medications [30],[31].

Before the introduction of atomoxetine, tricyclic antidepressants (TCAs) were a common alternative to the psychostimulants. However, few studies have compared the efficacy of these two drug classes and the association of TCAs with troublesome side effects, potential cardiotoxicity, and the risk of death in the event of overdose has taken TCAs out of favor [21],[24],[25]. The evidence base for other medications in the treatment of AD/HD is weak.

The most frequent troublesome side effects associated with the psychostimulant medications are reduced appetite, weight loss, and sleep difficulties [32],[33]. Compared to the pyschostimulants, atomoxetine has less-pronounced effects on appetite and sleep but is more likely to be associated with gastrointestinal upset [34]. Both the pyschostimulants and atomoxetine may be associated with slight increases in heart rate and blood pressure. Finally, the psychostimulants are controlled substances and concerns exist regarding the risk of pyschostimulant misuse and drug diversion. However, there is no clear evidence that pyschostimulant-treated AD/HD children abuse prescribed medication when they are appropriately diagnosed and carefully monitored [35].

Children treated with medication require careful clinical monitoring for side effects, clinical response, adherence, treatment acceptability, and dose adjustment. Treating clinicians must have a proficient understanding of the pharmacokinetic and pharmacodynamic properties of these medications, the potential for adverse events, and strategies for addressing emergent side effects. Medication monitoring should include: a baseline assessment followed by regular assessment of symptoms, function, side effects, adherence, and the emergence of comorbid disorders or other medical conditions; measurement of height, weight, blood pressure, and pulse; and provision of psychoeducation that addresses emerging questions or concerns from the patient or family.

There is limited empirical evidence (all from HICs) to inform the duration of treatment with pharmacological agents in AD/HD. Six- and eight-year follow-up data from the Multimodal Treatment Study of Children with AD/HD (MTA) failed to provide support for long-term medication treatment where care was provided in community settings without ongoing careful titration and monitoring [36]. Decisions about continuing or discontinuing medication should be individualized, with periodic discontinuations to assess need and benefit.

Finally, decisions regarding the use of pharmacological therapy should be informed by the level of impairment, symptom severity, availability of appropriately trained health personnel and support, accessibility to monitoring and follow-up services, acceptability to patient and the family, and cost. Pharmacologic treatment is not indicated if symptoms are mild, if there is minimal impairment or an unclear diagnosis, if there are inadequate services for close monitoring and follow-up, or if the use of medication is unacceptable to the patient or family.

### Structured Psychotherapies

Two of the largest studies of psychotherapies for the treatment of AD/HD ever conducted are the New York-Montreal Study and the MTA study [37]–[41]. In the New York-Montreal Study, 103 children with AD/HD (ages 7–9), who did not have comorbid conduct or learning disorders and who had responded to methylphenidate were allocated randomly to three groups for two years: (1) methylphenidate treatment alone; (2) methylphenidate combined with multimodal psychosocial treatment that included parent training and counseling, academic assistance, psychotherapy, and social skills training; or (3) methylphenidate plus attention control treatment that excluded specific aspects of the psychosocial intervention. Individual assessments were conducted on outcome measures such as academic and emotional status [39] and symptomatic improvement [40]. This study found no significant difference between the combined-treatment groups and the medication-only group. In the MTA study, 579 children aged 7.0 to 9.9 years with AD/HD were randomly assigned to four treatment groups (medication, behavioral treatment, combined treatment, and community care) [38]. The authors concluded that after the 24-month follow-up, medication was superior over the other individual treatment options and that there was no significant difference between the combined treatment and medication-only [41]. However, further analysis of the data concluded that patients with AD/HD and either comorbid disorders [42]–[44] or psychosocial stressors [45],[46] did benefit from adjunctive psychosocial interventions (parent training and behavioral interventions).

Several reviews have investigated whether psychosocial interventions are effective for treating children and adolescents with AD/HD (Table 1). A systematic literature review [47] and a meta-analysis that investigated 174 AD/HD treatment studies concluded that behavioral treatment is highly effective [48]. Reviews of cognitive-behavioral [49],[50] and family therapy [51],[52] interventions have yielded promising but inconclusive findings.

We were not able to locate any randomized controlled trials addressing the efficacy of structured psychotherapies for the treatment of AD/HD in LMICs. However, an Iranian study [53] reported that Parent Management Training (eight sessions, once a week for 1.5 hours) improved the behavior of 15 children with AD/HD and the general health of their parents. Similarly, an Indian study investigated the effectiveness of a program providing five training sessions to ten parents of boys aged 6–10 years with AD/HD. The training, which addressed attention-enhancing tasks and child behavior principles and techniques, had significant effects on the behavior of the children, and parent-reported study habits, pro-social behavior, and academic performance [54].

## Delivery of Effective Interventions

### Interventions to Increase Demand for Services

Similarly to the other disorders discussed in this series, stigma and a lack of awareness about mental health problems are important contributors to a relatively low demand for services for AD/HD in LMICs. In the case of AD/HD, however, the salience of these factors is magnified because AD/HD is most common among children and adolescents, an age group in which stigma and lack of awareness are particularly evident [55]. Among populations where stigma is high and knowledge levels low, efforts should be directed in the first instance to increasing general awareness and recognition of the disorder (Table 2). Once this goal has been achieved, attention may need to be focused on minority groups, inattentive type AD/HD, girls with AD/HD, and adult AD/HD. While specialists in mental health, education, and pediatrics should take a leading role in reducing stigma and increasing awareness, the potential contribution of community-based advocacy groups should not be ignored. School is a particularly important setting for stigma reduction and information raising activities. While we were unable to locate any reports that evaluated awareness or recognition programs for AD/HD specifically, Hoven et al. [56] assessed children, parents, and teachers before and after an awareness program to determine changes in knowledge, attitudes, and understanding of mental health in nine countries including Azerbaijan, Brazil, China, Georgia, Russia, and Uganda. The authors reported a positive change in awareness in all the countries and an increased willingness to discuss emotional problems freely.

**Table 2:** Delivery treatments for AD/HD.

Step	How	By Whom	In What Settings
**Increasing demand for services**	• Develop and implement mental health literacy programs for the general public, government, non-government and private sectors focused on increasing awareness and understanding of common mental disorders and addressing the stigma associated with them.• Develop and implement specific awareness programs for common child mental disorders specifically targeting the general public and community organizations that interface with children and adolescents.• Target awareness programs to increase recognition of specific subgroups once services are available and accessible to the most prevalent groups.	• Child and adolescent mental health specialists• Educational psychologists• Community pediatricians• Mental health advocacy groups	• Schools [56]• Faith-based organizations• Primary health care services• Social development offices• Traditional healing practices• General practices• Pediatric and psychiatric services
**Increasing access to AD/HD interventions**	• Create the necessary policy and legislative environment [57]• Ensure access to methylphenidate and other medications• Provide affordable and equitable services and referral pathways [9]• Develop services in rural settings• Encourage affordable local public transport to improve accessibility of services• Provide selected interventions in school settings [58]• Integrate child and adolescent mental health services with primary and traditional care systems• For adolescents, provide interventions ‘youth friendly’ settings• Offer incentives for child and adolescent mental health specialists to work outside of regional centers• Establish shorter and more direct training pathways to registration as a sub-specialist	• Child and adolescent mental health specialist teams• Community pediatric teams• General adult psychiatrists in regions without access to child and adolescent mental health services• Primary mental health nurses• Mental health advocacy groups• Politicians• Service planners and managers• Pharmacists	• Rural community clinics• Village health teams• General practice clinics• Community pediatric services• Schools• Youth clubs and centers
**Increasing the capacity of health care teams**	• Provide training, supervision, liaison and consultation for all providers or potential providers of services for people with AD/HD [9],[58]• Strengthen formal links between services based in cities and those in rural areas, partly using tele-psychiatry if feasible [57]• Establish shorter and more direct training pathways to registration as a sub-specialist	• Child and adolescent mental health specialist teams• Specialist and sub-specialist training institutions	• Rural community clinics• Village health teams• General practice clinics• Community pediatric services• General adult psychiatric services
**Improving recognition of AD/HD**	• Increase capacity of health care teams• Improve access to valid and reliable AD/HD rating instruments• Improve access to psychometric testing to evaluate overall intelligence and exclude specific learning disorders	• Child and adolescent mental health specialist teams• General adult psychiatrists in regions without access to child and adolescent mental health services• Educational psychologists• Community pediatricians	• Schools• Primary health care services• Social development offices• Traditional healing practices• General practices• Community pediatric practices• Institutional and alternative child-care settings
**Adapting AD/HD treatments to increase acceptability or reduce costs**	• Integrate mental health services into the education and health care systems [57]• Provide culturally acceptable and language-appropriate care [9]• Reduce racial/ethnic disparities in access to child and adolescent mental health services• Institute group psychotherapy interventions, including parent management training and social skills/anger management groups• Increase access to less stigmatizing, longer-acting stimulant medications that do not need to be taken during school hours• Develop nurse-led, specialist-supervised AD/HD clinics• Consider motivational enhancement therapy for adolescents	• Child and adolescent mental health specialist teams• Community paediatric teams• Child psychiatric nurse specialists• Primary mental health nurses and social workers• Educators and school counsellors	• Schools• Pre-schools• Community clinics• Community centers• Youth clubs/centers• General practice clinics• Community pediatric services• Child and adolescent mental health care services
**Developing community-based programs**	• Introduce classroom-based behavioral strategies• Encourage caretakers to use behavioral techniques and improve communication between them and children with the disorder• Persuade leaders of community-based programs to link people with the disorder with services that provide appropriate interventions• Institute early childhood development programs	• Child and adolescent mental health specialist teams• Community pediatric teams• General adult psychiatrists in regions without access to child and adolescent mental health services• Primary mental health nurses and social workers• Educators and school counselors	• Schools• Pre-schools• Community clinics• Community centers• General practice clinics• Rural outreach teams and village health teams• Community pediatric services• Child and adolescent mental health services
**Encouraging practice-based programs**	• Collaborative care	• Specialists in mental health• Non-professional health care workers	• Primary health care settings
**Addressing the impact of AD/HD on other health and social outcomes**	• Deliver services for people with AD/HD together with services for other disorders• Provide AD/HD services throughout the life span• Provide interventions for specific problems that are associated with AD/HD, such as anger management, family conflict, scholastic deficiencies and substance misuse	• Child and adolescent mental health specialist teams• Community pediatric teams• General adult psychiatrists in regions without access to child and adolescent mental health services• Mental health service managers and planners	• All settings where AD/HD interventions are delivered

### Interventions to Increase Access to Interventions

Important elements of a strategy for increasing access include advocacy for the development of new, or the revision of existing, policy and legislative frameworks that ensure the promotion of mental health and the development of services that are accessible, affordable, appropriate, and acceptable to the children, families, and communities they are meant to serve (Table 2). Other important elements include ensuring that evidence-based, cost-effective treatments are both accessible and consistently available and that appropriately trained, skilled, and experienced staff are available [57]. A crucial aspect of access to effective treatment for AD/HD is access to the psychostimulants and other pharmacological agents for which there is evidence of effectiveness. This may require that child and adolescent psychiatrists and pharmacists attend financial committees and other managerial structures to ensure that the appropriate psychopharmacological agents are available and affordable, that financing is sustainable, and that health and supply systems are reliable. Clearly, the inclusion of methylphenidate in national and transnational lists of essential drugs is crucial in facilitating such circumstances.

### Interventions to Increase the Capacity of Health Care Teams

People with AD/HD may interface with health providers in many settings. Thus, a broad range of health care providers may be potential targets of capacity development activities. Depending on the context, providers may include child and adolescent mental health specialists, pediatricians, general psychiatrists, general medical service providers, and other nonphysician health providers, particularly those who work closely with children and adolescents. Opportunities for continual learning and support through mentorship, supervision, and continuing education should be built into capacity development initiatives. Training content should be tailored to the needs of target groups but should be based on an understanding of AD/HD as a chronic, pervasive disorder requiring a holistic, multisectoral approach to management and should be provided as part of a broader curriculum that addresses the comorbid mental health issues associated with AD/HD. Ensuring that capacity development initiatives are accessible and available to target groups can be a challenge in any context, and how training is delivered may have to be tailored to best meet the needs of the recipients. General psychiatrists in rural areas might benefit from telepsychiatry to improve their prescribing practices, for example. Similarly, educators might benefit from continuing education seminars that focus on the identification of children with AD/HD and on classroom interventions, and pediatric primary health care nurses might benefit from supervised clinical practice followed by direct access to a nursing or medical specialist consultation.

### Interventions to Improve Recognition of AD/HD

Strategies to improve recognition of AD/HD should be implemented only once access to services has been ensured and health care providers have sufficient capacity to respond to an increased demand for interventions. There are three aspects to improving recognition: (1) awareness of the risk factors for AD/HD and the identification of persons at risk; (2) screening of persons at risk for ADHD; and (3) determination of a diagnosis of AD/HD. As mentioned above, there are many useful clinical instruments and rating scales for both screening and assessment of AD/HD. Depending on the context, informal health services, schools, faith-based organizations, and other community-based groups can be invaluable in the early recognition of risk factors for potential mental health problems including AD/HD. Where available, psychometric testing may also help to assess the extent to which low levels of intellectual functioning or deficits in academic skill contribute to the presentation. Clearly, such instruments, rating scales, and tests are most likely to produce optimum benefits when they are administered by people with the necessary skill, training, and facility in the language of the individuals who are being assessed.

### Interventions to Adapt Treatments to Increase Acceptability or Reduce Costs

Both methylphenidate and amphetamine, the best-studied and most cost-effective medicines for AD/HD, are available in long-acting preparations that require once daily dosing. Such dosing is often preferable to multiple daily dosing, particularly for school-aged children and adolescents, and should be made available where possible. Psychosocial interventions may be delivered in either individual or group format and may be offered in various settings including schools. Individual-level interventions, while having advantages over group-level interventions, are not always feasible because of the shortages of appropriately trained staff. While group-level interventions are implemented in many settings in both LMICs and HICs, there is a dearth of evaluations of such interventions, a gap that begs filling.

Integration of child and adolescent mental health services into the education and/or primary health care system can also increase the acceptability and affordability of AD/HD treatments [58]. There are numerous models for integrating mental health services into community and primary care that require the development of continuous care pathways linking different levels of the health system. One example is the development of nurse-led but specialist-supervised clinics. The strategy adopted will be highly dependent on the resources and infrastructure available in a particular setting.

### Interventions to Develop Community-Based Programs

The establishment of community-based initiatives in partnership with formal health services allows the development of a continuous network of care and support for children with AD/HD and their families. Such care and support is essential to achieving and maintaining optimal functioning and ameliorating the disability associated with AD/HD. Schools, where most children spend most of their time, can be the cornerstone of such programs. Educators can be helped to implement classroom-based behavioral interventions such as token economy systems in which children are rewarded immediately for desirable behavior with tokens that can be exchanged later for a reward, or response cost systems in which a “fine” is imposed for bad behavior. Other interventions that could be introduced include increasing teacher-to-child classroom ratios and breaking the school day into brief academic assignments interspersed with brief periods of physical exercise [58]. Community-based organizations can also be used to deliver parenting interventions and support for families and caregivers, to provide gatekeeper functions and public mental health education, and to assist with accessing medications for which there is evidence of effectiveness.

### Interventions to Encourage Practice-Based Programs

Collaborative care programs that are specific to a particular disorder (as opposed to more general strategies such as the integration of mental health care into the primary health care system) have been successfully implemented for depression [59]. Furthermore, there is evidence that with appropriate diagnostic and pharmacological training, both general practitioners and pediatricians can apply this intervention effectively in HICs for AD/HD [60],[61]. However, we were not able to locate any studies in which practice-based programs to address AD/HD have been implemented and evaluated in LMICs.

### Interventions to Address the Impact of AD/HD on Other Health and Social Outcomes

AD/HD is frequently comorbid with other mental health problems and is associated with a range of negative health and social outcomes. Thus, services for people with AD/HD should not be delivered in isolation from services for other disorders. Also, because most children with AD/HD will retain the diagnosis into adulthood or continue to be deleteriously affected by the symptoms associated with AD/HD, it is crucial that interventions are not terminated in adolescence, as frequently occurs. By continuing to receive intervention, the probability of long-term adverse consequences of the disorder is reduced. Finally, it is important to offer interventions for selected specific problems that are associated with AD/HD, such as anger management, family conflict, scholastic deficiencies, and substance misuse.

## Packages of Care for AD/HD in LMICs

AD/HD is a common developmental disorder that affects individuals throughout their lives and across all cultural contexts, and is associated with considerable social, psychological, and economic adversity. Recognition of the disorder can be improved by the use of screening instruments, some of which have been shown to have adequate psychometric properties in LMICs. Although there are few studies from LMICs that address the effectiveness of methylphenidate, the consistent and strong effects that have emerged from HICs suggest that this drug will be effective universally. In addition, although the evidence for structured psychotherapies is mixed, there is sufficient justification to include behavioral interventions in a package of care for AD/HD in LMICs (Table 3). However, given that most people worldwide with AD/HD will not receive interventions from a mental health specialist such as a psychiatrist or psychologist, it is essential that efforts to deliver this package of care in LMICs are not dependent on such specialists. There are risks associated with such an approach. For example, the quality of the interventions may be less than would have been the case if they were delivered by specialists. However, such risks need to be weighed against the certainty that services will be less accessible if they are delivered by specialists. Finally, it is crucial that this package of care for AD/HD form part of a more comprehensive package of services in which other disorders are also addressed.

**Table 3:** Packages of care for AD/HD.

Low-Resource Settings	High-Resource Settings
Screening of high-risk groups	Screening of high-risk groups or routine screening with confirmation of diagnosis by a skilled clinician
Psychoeducational interventions with caregivers	Psychoeducational interventions with caregivers
Methylphenidate	Methylphenidate and other medication
Behavioral interventions	Behavioral interventions

## Abbreviations

AD/HD: attention-deficit hyperactivity disorder
DISC-IV: Diagnostic Interview Schedule for Children Version IV
DSM: Diagnostic and Statistical Manual of Mental Disorders
HIC: high-income country
HKD: hyperkinetic disorder
ICD-10: International Classification of Diseases 10th edition
LMICs: low- and middle-income countries
MTA: Multimodal Treatment Study of Children with AD/HD
TCA: tricyclic antidepressant
